# Effects of Illegal Solid Waste Dumping on the Structure of Soil Bacterial Communities: A Case Study in China

**DOI:** 10.3390/toxics13010020

**Published:** 2024-12-27

**Authors:** Jialiang Chen, Lulu Zhang, Lang Song, Mai Ye, Lin Wang, Bin Fan, Bin Li, Zetao Yang, Rongzhou Jin, Pu Jia

**Affiliations:** 1 Guangdong Provincial Academy of Environmental Science, Guangzhou 510045, China; chenjialiang_email@163.com (J.C.); lulu5013@126.com (L.Z.); larrysoong@foxmail.com (L.S.); vein1980@163.com (M.Y.); diarmait15@163.com (L.W.); gzhufanbin@163.com (B.F.); lishite1988@163.com (B.L.); 15817065385@163.com (Z.Y.); 2 School of Life Sciences, South China Normal University, Guangzhou 510631, China; qingtianbiluo@163.com

**Keywords:** soil bacterial community, illegal dumping, solid waste, heavy metal, environmental damage confirmation, identification and assessment of environmental damage (IAED)

## Abstract

Illegal solid waste dumping is a significant factor contributing to environmental damage. In this study, 16S rRNA gene sequencing technology was used for the identification and assessment of environmental damage in an illegal dumping area in China, with the aim of confirming environmental damage through analyzing changes in the soil bacterial communities across slag, sewage sludge, and non-contaminated areas. The results indicate that the diversity of soil bacteria decreases with an increase in the degree of pollution. The illegal dumping of slag resulted in an increase in the relative abundance of *Firmicutes* and a decrease in the relative abundance of *Acidobacteriota*. Additionally, illegal dumping of sewage sludge resulted in an increase in the relative abundance of *Proteobacteria* and a decrease in the relative abundance of *Acidobacteriota*. The contents of Ni and Be in slag and Cu, Pb, and Cd in sewage sludge were key factors affecting bacterial community composition. The results reveal the effects of heavy metal pollution on the soil bacterial community structure and its environmental driving factors, thus expanding understanding in the context of management of the environmental damage caused by illegal dumping, as well as providing a perspective on the changes in the soil bacterial community, allowing for environmental damage confirmation.

## 1. Introduction

The environmental problem of solid waste has gradually become a worldwide issue. As the global population grows and consumption levels rise, solid waste continues to increase, exacerbating the triple planetary crises [1]—climate change [2,3], pollution [4,5], and nature and biodiversity loss [6,7]. The effective treatment of solid waste requires not only advanced technology and adequate financial support, but also worldwide synergistic co-operation, which has become an important issue in modern global governance [8].

At the same time, the improper treatment, disposal, and even illegal dumping of solid waste poses a significant environmental concern [9,10,11,12]. Although the Ministry of Ecology and Environment (MEE) of China clearly requires that solid waste must not be dumped, stacked, discarded, or scattered without authorization [13], illegal solid waste dumping continues to occur, posing a significant threat to the environmental quality and ecological integrity of the soil [14,15]. The number of environmental damage compensation incidents has shown a significant increase, with soil pollution incidents accounting for the largest share, thus becoming a prominent and challenging aspect of environmental litigation [16].

Many countries and regions, including the USA, Japan, Australia, and the EU, have paid attention to the identification and assessment of environmental damage (IAED) [17,18,19,20]. They have conducted extensive theoretical research and implemented practical applications, which have been accepted in accordance with local developmental differences. These efforts have led to the definition of connotations, goals, objects, and contents, resulting in the establishment of various liability systems. Since 2011, China has been gradually implementing the IAED to deal with environmental crimes [21]. IAED involves the investigation and analysis of the causal relationship between the acts of environmental pollution or ecological damage, the assessment of the scope and extent of the environmental damage resulting from the incident, the determination of environmental restoration to baseline conditions, and the compensation of restoration measures and damage costs incurred during the process [22]. Environmental damage confirmation constitutes a pivotal aspect of IAED.

Compared to other forms of environmental pollution, soil pollution is concealed and accumulative, making it more difficult to investigate its causes and determine liability. As a result, the procedures and methodologies used to identify and quantify environmental damages are more complicated [23]. The soil serves as a microenvironment that is conducive to the growth and proliferation of bacterial communities, with its physical and chemical properties influencing the structure of these communities [24,25,26,27,28]. Studies have shown that heavy metals derived from human production activities enter the soil directly or indirectly in liquid (mainly as water), gaseous, and solid forms, which cannot be decomposed by soil bacteria and can be transformed into more toxic compounds [29,30,31,32]. Bacterial responses to changes and perturbations in the soil environment are both rapid and sensitive [33,34,35,36]. Therefore, the structure and diversity of bacterial communities can serve as indicators of changes in soil pollution levels and ecological function, as well as provide insights into the toxicity and potential effects of pollutants [37,38,39]. High-throughput sequencing technology based on 16S rRNA has been widely used to analyze the characteristics of bacterial communities and to reveal the effects of contaminants on bacterial community structure and diversity [40,41,42]. However, at present, environmental damage confirmation in IAED is mainly based on examination of the concentrations of heavy metals or organic compounds, while research on the application of bacterial community structure analyses remains limited. Furthermore, there is a lack of practical IAED approaches regarding illegal dumping behaviors at the bacterial level.

Given the frequent occurrences of illegal solid waste dumping and the resulting environmental hazards, this study examined a representative incident in the Greater Bay Area of China as an example of IAED. Soil samples were collected from areas where slag and sewage sludge had been dumped for about two decades, as well as from a non-contaminated area. Through an analysis of soil characteristic pollutants and high-throughput sequencing, the effects and correlations of different solid waste pollution characteristics on soil bacterial community structures were investigated. Furthermore, we explored the potential applications of this approach in the IAED context. This study reveals the environmental damage caused by illegal dumping of solid wastes from a microbial perspective and provides valuable insights for the improvement and refinement of IAED technology systems.

## 2. Materials and Methods

### 2.1. Survey Regions

**Figure 1:**
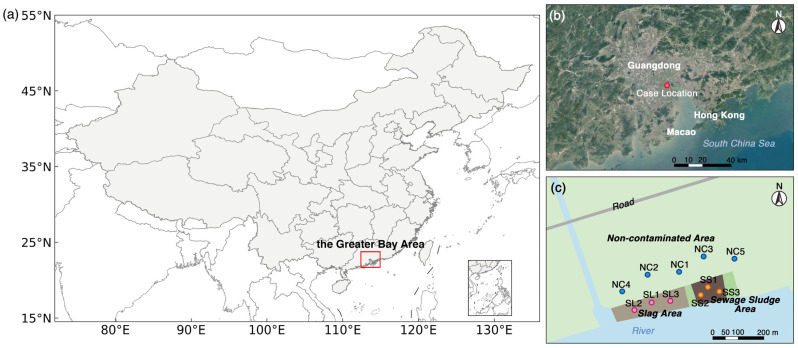
Sampling site location. (**a**) The location of the Greater Bay Area in China; (**b**) the case location in the Greater Bay Area; and (**c**) the distribution of sampling sites. Abbreviation: slag area (SL1–SL3), sewage sludge area (SS1–SS3), and non-contaminated area (NC1–NC5).

The illegal solid waste dumping case investigated in this study occurred in the Greater Bay Area (i.e., the Pearl River Delta Region), located in southern China (Figure 1a,b). The Greater Bay Area stands out as one of China’s most economically developed urban agglomerations, exemplifying the complex interplay between economic growth and environmental preservation. The area is characterized by flat topography, and the soil types are mainly ferralitic and anthropic soils. It experiences a subtropical monsoon climate, with an average annual temperature of 22.6 °C and an average annual precipitation of 1920.4 mm.

The sampling site covered an area of 42,000 m^2^. Prior to 2002, it served as a fishpond and wasteland. From 2002 to 2015, it underwent development for building construction and was utilized for the pre-treatment and storage of recycled solid waste. Since 2015, the buildings on this site have been demolished, and there are plans for it to be repurposed as an embankment protection green space and road land.

The IAED identified two types of solid waste dumped in the study site: slag covering an area of ~20,000 m^2^, with an estimated weight of ~62,000 t; and sewage sludge covering an area of ~6000 m^2^, with an estimated weight of ~18,000 t. The IAED concluded that illegal solid waste dumping necessitated consideration of the resource value of ~80,000 m^3^ of damaged soil and ~5000 m^3^ of damaged groundwater, resulting in an environmental damage cost of ~50 million yuan (equivalent to ~6.9 million U.S. dollars). This cost encompassed expenses associated with the cleaning and disposal of solid waste, as well as the value of the environmental damage to the soil and groundwater.

### 2.2. Collection of Solid Waste and Soil Samples

Sample collection of solid waste and soil accompanying IAED was carried out in July of 2022 (Figure 1c). A total of 11 sampling sites were selected in 3 types of areas; namely, slag (3 sites, SL1–SL3), sewage sludge (3 sites, SS1–SS3), and non-contaminated (5 sites, NC1–NC5) areas.

For solid waste sampling, a total of 6 samples were collected from the slag area (SL1–SL3) and the sewage sludge area (SS1–SS3). Using sterile shovels, all samples were collected randomly from the dumping layer and placed into ziplock bags (~1000 g per sample) and brown glass bottles (~500 g per sample) and stored at 4 °C for subsequent determination of the solid waste characteristic pollutants. The detailed sampling and preparation methods for the solid waste samples all complied with the MEE standards [43].

For soil sampling, a 1 m × 1 m quadrat was demarcated at each site, and a five-point sampling method was adopted to collect the surface soil at a depth of 0–10 cm. A total of 11 soil samples were collected in this study. All samples were collected with a sterile shovel and placed into ziplock bags (~500 g per sample). Then, the soil samples were kept on ice and transported to the laboratory as soon as possible. Each sample was divided into two parts, except the samples NC4 and NC5, which were used only for the determination of the soil characteristic pollutants and calculation of the baseline value of the undamaged environment. One part was naturally dried, passed through a 100-mesh sieve to remove the plant debris and rocks, and stored at 4 °C for subsequent determination of soil characteristic pollutants. The other part was immediately stored at −80 °C for further DNA extraction and high-throughput sequencing of the microbiome. The detailed sampling and preparation methods for the soil samples followed the MEE standards [44].

### 2.3. Determination of Characteristic Pollutants

The test methods for solid waste pollutants referred to the MEE standards [45,46,47,48]. High-resolution time-of-flight mass spectrometers were utilized to screen characteristic solid waste pollutants, with heavy metals determined via inductively coupled plasma-mass spectrometry (ICP-MS 7900, Agilent Technologies, Singapore), and semi-volatile organic compounds and volatile organic compounds analyzed via gas chromatography-mass spectrometer (GC-MS 8860-5977B, Agilent Technologies, Santa Clara, CA, USA). Based on the screening results, the characteristic pollutants of solid waste were identified as Cu, Ni, Zn, Pb, Be, and Cd, and organic pollutants were not detected. These pollutants were then used as references to determine the corresponding characteristic pollutants in the soil, considering the causal relationship of the pollution incident.

The test methods for soil pollutants referred to the MEE standards [49,50,51,52]. Soil pH was determined using an acidimeter (PHS-3C, INESA, Shanghai, China), while heavy metal elements were analyzed via atomic absorption spectroscopy. Specifically, Cu, Ni, and Zn were measured using 240FS AA atomic absorption spectrophotometer (Agilent, Petaling Jaya, Malaysia); Pb was measured using 280FS AA atomic absorption spectrophotometer (Agilent, Petaling Jaya, Malaysia); Be was analyzed using 240Z AA atomic absorption spectrophotometer (Agilent, Petaling Jaya, Malaysia); and Cd was assessed using 280Z AA atomic absorption spectrophotometer (Agilent, Petaling Jaya, Malaysia).

### 2.4. DNA Extraction and Amplicon Sequencing

DNA was extracted from a 350 mg soil sample using the TS96 Magnetic Soil DNA Kit (Tiangen Biotech, Beijing, China) [53,54], following the manufacturer’s instructions. The concentration of extracted DNA was measured using the Qubit dsDNA HS Assay Kit and a Qubit 4.0 Fluorometer (Invitrogen, Thermo Fisher Scientific, Waltham, MA, USA). Subsequently, the V3-V4 region of the 16S rRNA gene was amplified from the genomic DNA of each sample using universal primers 338F (5′-ACTCCTACGGGGAGGCAGCA-3′) and 806R (5′-GGACTACHVGGGTWTCTAAT-3′). Both forward and reverse 16S primers carried sample-specific Illumina index sequences for deep sequencing. The PCR was performed in a total reaction volume of 10 µL: DNA template 5–50 ng, 0.3 µL of Vn F (10 µM), 0.3 µL of Vn R (10 µM), KOD FX Neo Buffer 5 µL, dNTP (2 mM each) 2 µL, KOD FX Neo 0.2 µL, and ddH_2_O up to 10 µL. VN F and VN R were selected according to the amplification area. The PCR cycling conditions included initial denaturation at 95 °C for 5 min, followed by 25 cycles of denaturation at 95 °C for 30 s, annealing at 50 °C for 30 s, extension at 72 °C for 40 s, and final extension at 72 °C for 7 min. The PCR amplicons were purified using Agencourt AMPure XP Beads (Beckman Coulter, Indianapolis, IN, USA), and quantified again using the Qubit dsDNA HS Assay Kit and a Qubit 4.0 Fluorometer. After quantification, amplicons were pooled in equal amounts for library construction and sequenced on an Illumina NovaSeq 6000 platform (Illumina, San Diego, CA, USA). Raw sequences were deposited in the NCBI Sequence Read Archive with the accession number PRJNA1122348 (https://www.ncbi.nlm.nih.gov/bioproject/PRJNA1122348, accessed on 11 Jnue 2024).

### 2.5. Data Analyses

Soil environmental baseline values were referenced from the MEE standard [55]. Statistical calculations of baseline values were calculated using data from the non-contaminated area. For data following a normal distribution, the upper 90th percentile reference limit was determined as the arithmetic mean plus 1.65 times the standard deviation of the non-contaminated area data. For data not following a normal distribution, the 90th percentile of the non-contaminated area data was used as the baseline.

High-throughput sequencing analysis and mapping were conducted using Biomarker Cloud (Biomarker Technologies, Beijing, China). Initially, raw data underwent quality filtering using Trimmomatic (Version 0.33) [56] in order to remove low-quality data. Primer sequences were identified and eliminated using Cutadapt (Version 1.9.1) [57]. Subsequently, paired-end reads obtained from the previous steps were assembled using USEARCH (Version 10.0) [58], followed by chimera removal using UCHIME (Version 8.1). Finally, high-quality sequences were obtained for subsequent analysis.

The sequencing results were similar at a level greater than 97%. OTU clustering analysis of sequences was performed using USEARCH (Version 10.0) [58], and the data were denoised after quality control using the DADA2 method in QIIME2 (Version 2020.06) [59] in order to resolve α-diversity indices (Shannon–Wiener, Simpson, ACE, Chao1). Principal coordinate analysis (PCoA) of the samples was plotted based on R studio 4.0.3 (R Development Core Team, 2019), and the correlation analysis between the characteristic pollutants and bacterial community composition was performed via redundancy analysis (RDA) and heatmap analysis.

## 3. Results

### 3.1. Soil Pollution Status

According to the obtained results (Table 1), the soil samples from the slag, sewage sludge, and non-contaminated areas were all weakly alkaline, with pH values ranging from 8.05 to 8.37. Heavy metal concentrations in soil samples from both the slag and sewage sludge areas exceeded the baseline values, to varying degrees: soil samples from the slag area exceeded the baselines by 0.23–1.05 times for Cu, Ni, and Zn, while Pb, Cd, and Be did not exceed the baseline; meanwhile, soil samples from the sewage sludge area exceeded the baseline by 0.04–4.69 times for Cu, Ni, Zn, Pb, Cd, and Be. However, none of the characterized pollutants mentioned above exceeded the soil environmental quality standards of China [51]. Under the influence of the illegal dumping of solid waste, both the slag area and the sewage sludge area exhibited evident heavy metal pollution in the soil. Comparatively, the polluted condition in the sewage sludge area was slightly more severe than that in the slag area.

### 3.2. Sequencing Results and α-Diversity Indices

When the number of test sequences for each sample reached 50,000, the sequencing coverage reached 99.97% and greater, indicating sufficient sequencing depth to encompass most bacteria. Regarding OTUs (operational taxonomic units) and α-diversity indices (ACE, Chao1, Shannon–Wiener, Simpson), the non-contaminated area exhibited the highest number of OTUs, followed by the slag area, while the lowest was observed in the sewage sludge area (Table 2). Additionally, the α-diversity indices (ACE, Chao1, Shannon–Wiener, Simpson) in the slag and sewage sludge areas were lower than those in the non-contaminated area. This suggests that heavy metal pollution substantially decreased bacterial biomass, and that the diversity of soil bacteria declined with increasing pollution levels. Moreover, differences in OTUs and α-diversity indices were observed between the slag and sewage sludge areas: the slag area exhibited relatively high OTUs, ACE, and Chao1 indices, with values of 1397 ± 375, 1400.72 ± 374.13, and 1397.50 ± 374.73, respectively, whereas the sewage sludge area showed relatively high Shannon–Wiener and Simpson indices, with values of 7.67 ± 0.17 and 0.986 ± 0.001, respectively.

### 3.3. Bacterial Community Structure

**Figure 2:**
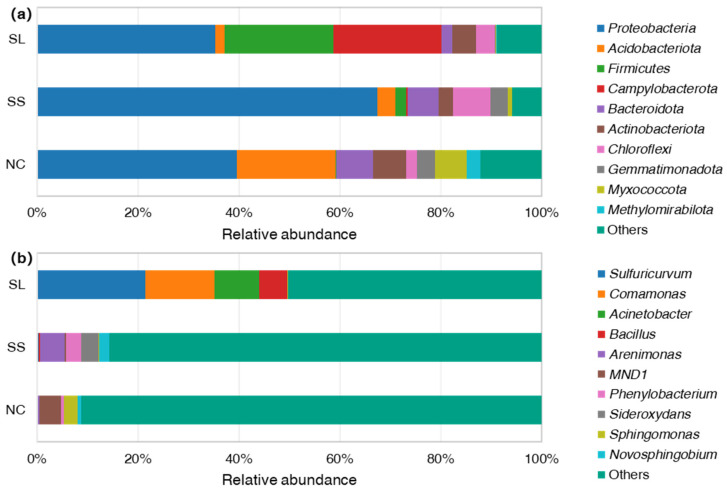
Relative abundance of soil bacteria at different taxonomic levels: relative abundance of soil bacteria at (**a**) phylum level and (**b**) genus level. Abbreviation: SL, slag area; SS, sewage sludge area; NC, non-contaminated area. Colors represent different bacterial taxa.

According to the results of the OTU analysis, taxonomic analysis was performed on the sample data at both the phylum and genus levels. Figure 2 shows a histogram illustrating the relative abundance of taxa at these levels among soil bacteria. Only the top 10 dominant taxa with relative abundance are displayed in the figure, while the remaining taxa are grouped under “Others.” The subsequent analysis centers on the dominant taxa at phylum and genus levels, both of which exhibited relative abundances exceeding 5% and 3%, respectively.

At the phylum level (Figure 2a), the dominant taxa in the slag area included *Proteobacteria* (35.36%), *Firmicutes* (21.55%), and *Campylobacterota* (21.47%). In the sewage sludge area, the only dominant phylum was *Proteobacteria* (67.48%). In the non-contaminated area, the dominant taxa included *Proteobacteria* (39.68%) and *Acidobacteriota* (19.43%). It was observed that *Proteobacteria* is the predominant phylum in the study area, with its dominance being particularly pronounced in the sewage sludge area.

At the genus level (Figure 2b), there were differences in the dominant taxa found in the slag, sewage sludge, and non-contaminated areas. In the slag area, the dominant taxa included *Sulfturicuryum* (21.44%), *Comamonas* (13.73%), *Acinetobacter* (8.92%), and *Bacillus* (5.50%). In the sewage sludge area, the dominant taxa included *Arenimonas* (4.99%), *Sideroxydans* (3.43%), and *Phenylobacterium* (3.04%). In the non-contaminated area, the dominant taxa included *MND1* (4.23%).

**Figure 3:**
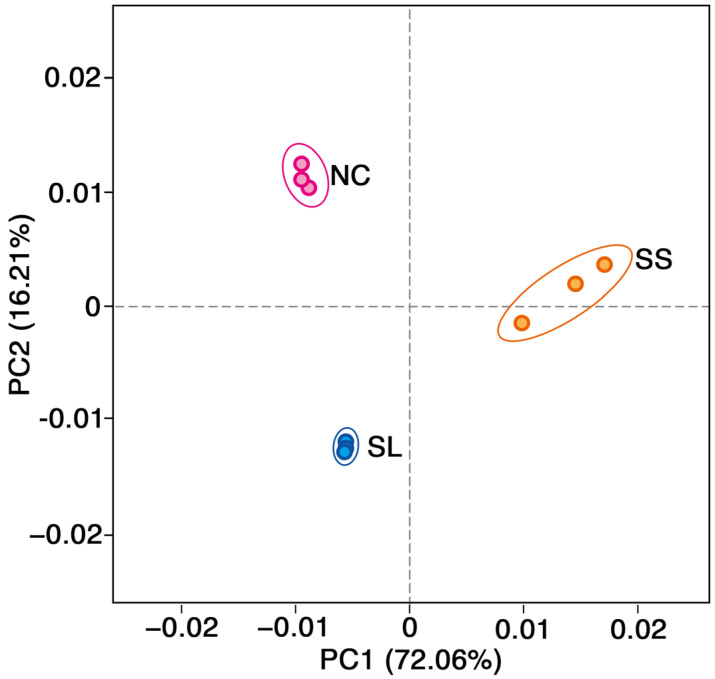
Principal coordinate analysis (PCoA) using the Bray–Curtis distance, depicting differences in soil bacterial communities in different areas and the 95% confidence interval ellipse was drawn. Abbreviation: SL, slag area; SS, sewage sludge area; NC, non-contaminated area.

The principal coordinate analysis (PCoA) revealed differences in the bacterial community composition among the soil habitats in the slag, sewage sludge, and non-contaminated areas (Figure 3). The PCo1 and PCo2 axes explained 64.25% and 27.83% of the total variation, respectively. The distribution within the slag area group was relatively clustered, indicating less variation in soil bacterial community structure within this area. Conversely, the distribution within the non-contaminated area groups showed a certain degree of dispersion, with the sewage sludge area group exhibiting more dispersed distribution, suggesting greater variation in soil bacterial community structure within these areas compared to the slag area. These findings revealed differences in bacterial community structure composition among the three soil habitat types.

### 3.4. Correlation Between Bacterial Communities and Characteristic Pollutants

**Figure 4:**
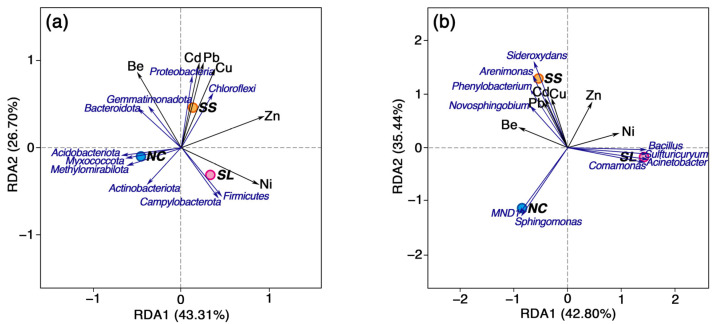
Redundancy analysis (RDA) of soil bacterial communities and characteristic pollutants at different levels: (**a**) phylum level and (**b**) genus level. Abbreviation: SL, slag area; SS, sewage sludge area; NC, non-contaminated area.

The top 10 dominant taxa at the phylum and genus levels were analyzed alongside characteristic pollution indices (Figure 4), and the results revealed distinct responses of the three dominant taxa to the characteristic pollution indices. In the slag area, Ni exhibited a positive correlation with the dominant taxa, while Be showed a negative correlation. The primary dominant taxa identified were *Firmicutes*, *Campylobacterota*, *Acinetobacter*, *Sulfturicuryum*, *Bacillus,* and *Comamonas*. Conversely, in the sewage sludge area, Cu, Pb, and Cd displayed positive correlations with the dominant taxa, which were primarily represented by *Proteobacteria*, *Choloroflexi*, *Sideroxydans*, *Arenimonas*, *Phenylobacterium,* and *Novosphingobium*. In the non-contaminated area, Ni and Zn exhibited negative correlations with the dominant taxa, which mainly comprised *Acidobacteriota*, *Methylomirabilota*, *Myxococcota*, *MND1,* and *Sphingomonas*.

**Figure 5:**
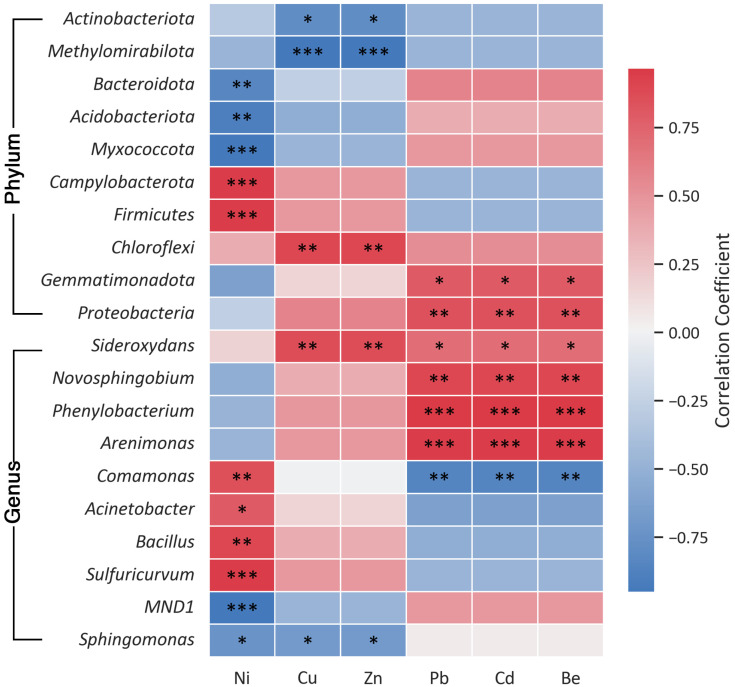
Heatmap of soil bacterial communities and characteristic pollutants. Asterisks represent significance levels: ***, *p* < 0.001; **, *p* < 0.01; *, *p* < 0.05.

The significance of the correlations was further analyzed using a Pearson correlation analysis heatmap (Figure 5), which revealed that the correlations between bacterial community structures and characteristic pollutants varied. Different bacterial communities could demonstrate either similar or opposing significant correlations with the same characteristic pollutants. For instance, *Myxococcota* displayed a significant negative correlation with Ni, which contrasted with the correlation patterns observed for *Firmicutes* and *Campylobacterota*. Conversely, the significant correlation patterns observed for *Phenylobacterium* and *Arenimonas* remained consistent across all heavy metals.

## 4. Discussion

### 4.1. Effects of Characteristic Pollutants on Soil Bacterial Diversity

In this study, the structure of damaged soil bacterial communities and their changes were studied for IAED. The comparison results of OTUs and α-diversity indices showed that the soil bacterial diversity was the highest in the non-contaminated area, while heavy metal pollution resulting from illegal solid waste dumping reduced the bacterial biomass in the slag and sewage sludge areas. The diversity of soil bacteria decreased with an increase in the pollution degree—a conclusion consistent with previous studies [60,61,62,63]—providing valid evidence for confirmation of environmental damage. The total contents of heavy metals such as Cd, Zn, Cu, Pb, Ni, and Be in soil contaminated with heavy metals can reduce bacterial biomass and diversity [64,65,66,67]. The differences in bacterial diversity among the three soil habitats may be related to differences caused by long-term heavy metal pollution in soil ecosystems [37,68,69].

The Shannon–Wiener index results obtained in this study differed from the OTUs, ACE, and Chao1 indices, with the Shannon–Wiener index being higher in the sewage sludge area compared to the slag area. Previous studies have also noted that the Shannon–Wiener index is not always the lowest in samples with high heavy metal concentrations, suggesting that the impact of pollution levels on bacterial diversity may be non-linear [7,8,9,10,11,12,13,14,15,16,17,18,19,20,21,22,23,24,25,26,27,28,29,30,31,32,33,34,35,36,37,38,39,40,41,42,43,44,45,46,47,48,49,50,51,52,53,54,55,56,57,58,59,60,61,62,63,64,65,66,67,68,69,70,71,72]. One or more heavy metals were found to contribute to the dominant taxa in the slag and sewage sludge areas, which implies that long-term soil heavy metal pollution may lead to bacterial resistance and the proliferation of dominant taxa [73,74,75].

### 4.2. Effects of Characteristic Pollutants on Dominant Taxa

The comparison of different illegal solid waste dumping behaviors revealed that variations in characteristic pollutants resulted in distinct effects on bacterial community structure [74,76]. At the phylum level, the illegal dumping of slag led to an increase in the relative abundance of *Firmicutes*, while decreasing the relative abundance of *Acidobacteriota*; meanwhile, the illegal dumping of sewage sludge resulted in an increase in the relative abundance of *Proteobacteria* and a decrease in the relative abundance of *Acidobacteriota*. Additionally, *Proteobacteria* emerged as the dominant phylum, consistent with findings from numerous previous studies on heavy metal pollution [77,78,79,80].

The root cause of the differences in bacterial community structure lies in the varied responses of bacterial taxa to changes in environmental factors [81,82]. In areas affected by illegal solid waste dumping, the relative abundance of bacteria sensitive to heavy metals drastically decreases, while those resistant to heavy metals may proliferate due to their rapid response and adaptability to heavy metal stress, thereby altering the soil community structure. *Firmicutes* may exhibit better adaptability to heavy metal pollution in slag areas, as indicated by their similar responses in the correlation analysis. *Proteobacteria*, with a relative abundance exceeding 60%, appears to demonstrate better adaptability under heavy metal pollution stress. Conversely, *Acidobacteriota* is notably sensitive to heavy metal stress, particularly to Ni.

### 4.3. Correlation Analysis Between Characteristic Pollutants and Bacterial Communities

Due to the illegal dumping behavior of solid waste approximately 20 years ago, the study area exhibited evident and intricate heavy metal pollution, including Cu, Ni, Zn, Pb, Cd, and Be. Our study revealed that soil heavy metal pollution was more severe in the sewage sludge area when compared to the slag area. Linking the characteristics of illegal dumping incidents to the confessions of offenders, we traced the source of the solid waste. Combining the incident information and characteristic pollutants, it is speculated that the slag originated from industrial incinerators, while the sewage sludge originated from urban domestic sewage treatment plants treating both domestic sewage and industrial wastewater. The heavy metals in slag are more likely to exist in stable residual states, whereas those in sewage sludge may predominantly exist as a mix of reducing and oxidizing components, along with other unstable states [83,84,85]. The mobility of heavy metals makes them more susceptible to transfer into the soil under the influence of rainfall leaching, resulting in greater accumulation of pollutants in the soil environment of the sewage sludge area. We recommend implementing targeted measures for the timely removal of pollutants and effective remediation of the soil environment in areas of historical illegal dumping incidents and landfilling of industrial solid waste.

The PCoA results revealed the differences in bacterial communities and dominant bacteria among the three soil habitats. Combining the outcomes of the RDA and the heatmap correlation analysis, it was observed that the illegal dumping of slag had the most pronounced impact on Ni, leading to increases in the relative abundance of *Firmicute* and *Campylobacteriota* at the phylum level, possibly due to the promotive effect of Ni. Conversely, it decreased the relative abundance of *Acidobacteriota*, which could be attributed to the inhibitory effect of Ni [64]. On the other hand, the illegal dumping of sewage sludge elevated the relative abundance of *Proteobacteria* at the phylum level, potentially due to the promotive effects of Pb, Cd, and Be, while diminishing the relative abundance of *Acidobacteriota*, possibly due to the inhibitory effect of Zn, similar to previous studies [67,75]. According to the correlation analysis results, *Proteobacteria* may exhibit some resilience in polluted areas [86]. Understanding the variations in heavy metal concentrations and bacterial communities across different soil habitats is essential for the identification of environmental damage and the development of effective functional restoration strategies for soil ecosystems [63,68,87].

### 4.4. Consideration on the Application of Bacterial Community Research in the Identification of Environmental Damage

IAED work typically relies on baseline comparisons for the confirmation of environmental damage. This process mainly refers to the series of standards of the Technical Guide for IAED [22,55,88,89]. Although biodiversity investigations—including community characteristics and biodiversity—are mentioned in the general program’s survey link, this information is not highlighted as key evidence in damage determination, and no such applications have been reported. However, this study demonstrated that heavy metal pollution resulting from illegal solid waste dumping reduced soil bacterial biomass and diversity, thus altering bacterial community structures. This finding can be considered effective evidence confirming the environmental damage to the soil caused by illegal dumping.

In recent years, China has made substantial efforts to establish a technical system for identifying and assessing ecological and environmental damage, with ongoing improvements [90]. Therefore, it is recommended to incorporate studies on bacterial community structure and changes into ecological environment damage identification practices, particularly in scenarios where conventional physical and chemical indicators show light pollution degrees or damage identification is challenging. This approach can provide a better indication of pollution and allow for effective damage determination, contributing to the construction and enhancement of environmental damage identification and assessment standard systems.

## 5. Conclusions

This study revealed the impacts of illegal solid waste dumping on soil bacterial community diversity and composition. Compared to the non-contaminated area, the illegal dumping of slag resulted in an increase in the relative abundance of *Firmicutes*, while decreasing the relative abundance of *Acidobacteriota*. Additionally, illegal dumping of sewage sludge resulted in an increase in the relative abundance of *Proteobacteria* and a decrease in the relative abundance of *Acidobacteriota*. We observed that environmental pollution resulting from illegal solid waste dumping decreased the number of soil bacteria, with the diversity of soil bacteria declining as the pollution level increased. In the slag area, the key factors affecting the difference in bacterial community composition were Ni and Be, while Cu, Pb, and Cd were key factors in the sewage sludge area. The correlations between characteristic pollutants and bacterial taxonomic groups varied. Overall, the results of this study shed light on the ecological damage to the soil microenvironment caused by illegal solid waste dumping, suggesting that information relating to the microbial community could be applied in the practice of IAED.

## Figures and Tables

**Table 1:** Soil characteristic pollution status.

Sampling Area	pH	Cu (mg/kg)	Ni (mg/kg)	Zn (mg/kg)	Pb (mg/kg)	Cd (mg/kg)	Be (mg/kg)
Slag area	8.33	87	86	442	52	0.76	1.79
Sewage sludge area	8.13	404	55	498	120	2.42	2.99
Non-contaminated area	8.24	52	36	150	59.80	0.82	2.67
Baseline value *	/	71	42	216	76	0.99	2.88
Risk control standard **	/	2000	150	10,000	400	20	15

* Baseline value was calculated using data from the non-contaminated area. For data following a normal distribution, the upper 90th percentile reference limit was determined as the arithmetic mean plus 1.65 times the standard deviation of the non-contaminated area data. For data not following a normal distribution, the 90th percentile of the non-contaminated area data was used as the baseline. ** Risk control standard referred to MEE standard [51].

**Table 2:** Diversity indices of soil bacterial communities. Different letters following the values indicate significant differences among samples (*p* < 0.05).

Sampling Area	Number of OTUs	Shannon–Wiener	Simpson	ACE	Chao1
Slag area	1397 ± 375 b	6.86 ± 0.81 b	0.960 ± 0.019 b	1400.72 ± 374.13 b	1397.50 ± 374.73 b
Sewage sludge area	1103 ± 124 b	7.67 ± 0.17 b	0.986 ± 0.001 a	1107.31 ± 123.82 b	1103.03 ± 123.69 b
Non-contaminated area	2099 ± 52 a	9.97 ± 0.06 a	0.998 ± 0.001 a	2102.12 ± 52.43 a	2099.13 ± 52.19 a

## Data Availability

Raw sequences were deposited in the NCBI Sequence Read Archive with the accession number PRJNA1122348 (https://www.ncbi.nlm.nih.gov/bioproject/PRJNA1122348, accessed on 11 June 2024).
